# Pathological Appearances Presented in the Bodies of Those Who Have Died Insane

**Published:** 1831-04-01

**Authors:** 


### LUNATIC HOSPITAL OF TURIN.

### PATHOLOGICAL APPEARANCES PRESENTED IN THE BODIES OF THE INSANE DEAD.

1. *Case of Cheerful Monomania.* The dura mater much injected, and adhering in many places to the pia mater—the latter softened—the brain pale and rather softer than natural—cerebral convolutions effaced—right ventricle full of water—cerebellum and spinal marrow sound—lungs disorganized—stomach and intestines livid, with gangrenous spots—liver softened.

2. *Melancholic Monomania with tendency to Suicide.* Membranes of the brain gorged with blood—no other unusual appearance in the head or chest—small intestines inflamed, as was the whole mucous membrane of the digestive tube, from the mouth downwards—many aphthæ in this track—liver enlarged and indurated—biliary concretions in the gall-bladder—spleen enlarged and indurated.

3. *Mania with Chorea.* Effusion under the arachnoid—cerebral substance hard and much injected—sanguineous effusion into the spinal canal, with traces of inflammation along the spinal marrow—all the other organs sound.

4. *Mania which terminated in Idiocy.* Some points of gangrenous aspect in the small intestines—brain very much softened, with much serum in the ventricles—nothing unusual in the other organs.

5. *Suicidal Mania.* Meninges and cerebral substance highly injected — heart large—traces of phlogosis in the digestive tube, especially in the duodenum and jejunum, whose parietes were thickened.

6. *Mania with Pride.* Meninges thickened and injected, having the appearance of parchment — strong adhesions between the two hemispheres—brain itself much injected — intestinal tube slightly inflamed.

7. *General Mania.* Much effusion between the membranes of the brain—pia mater inflamed even to suppuration —cerebellum sound, as were the thoracic and abdominal viscera.

8. *Melancholia.* Considerable inflammation in the small intestines — cerebral meninges injected — the other organs sound. — *Annale Universali de Medicina,* 1830.

These appearances only prove that the insane will not die, no more than the sane of mind, without some corporealdisease whichalmost alwaysleaves traces of its existence in the dead body. But they throw little light on the cause of the maniacal or monomaniacal phenomena. We can hardly doubt, indeed, that the functions of the mind can be disturbed, without some antecedent or cotemporaneous disturbance of a corporeal structure—and we may suspect the seat of the functional disturbance in the early part of the malady, by finding traces of altered structure after death. Yet it is only surmise, and not proof. Suppose the primary irritation which disturbs the judgment or reason of an individual be seated in the digestive tube : — that irritation may have ceased long before death, but long after the irritation was seen established in the brain or its membranes, where we find the traces of death on dissection. To one conclusion we may safely come, namely, that in every kind of mania, there is a bodily irritation somewhere, and that the brain or organ of thought must become either primarily or secondarily affected, before the phenomena of insanity shew themselves.

